# Marine Polysaccharides in Pharmaceutical Applications: Fucoidan and Chitosan as Key Players in the Drug Delivery Match Field

**DOI:** 10.3390/md17120654

**Published:** 2019-11-21

**Authors:** Ana Isabel Barbosa, Ana Joyce Coutinho, Sofia A. Costa Lima, Salette Reis

**Affiliations:** LAQV, REQUIMTE, Departamento de Ciências Químicas, Faculdade de Farmácia, Universidade do Porto, Rua de Jorge Viterbo Ferreira, 228, 4050-313 Porto, Portugal; anabarbosa.cc90@gmail.com (A.I.B.); ana.c.joyce@gmail.com (A.J.C.); shreis@ff.up.pt (S.R.)

**Keywords:** anticancer compounds, nanoparticles, ionic crosslinking method, polyphenolic compounds, polyelectrolyte self-assembly method

## Abstract

The use of marine-origin polysaccharides has increased in recent research because they are abundant, cheap, biocompatible, and biodegradable. These features motivate their application in nanotechnology as drug delivery systems; in tissue engineering, cancer therapy, or wound dressing; in biosensors; and even water treatment. Given the physicochemical and bioactive properties of fucoidan and chitosan, a wide range of nanostructures has been developed with these polysaccharides per se and in combination. This review provides an outline of these marine polysaccharides, including their sources, chemical structure, biological properties, and nanomedicine applications; their combination as nanoparticles with descriptions of the most commonly used production methods; and their physicochemical and biological properties applied to the design of nanoparticles to deliver several classes of compounds. A final section gives a brief overview of some biomedical applications of fucoidan and chitosan for tissue engineering and wound healing.

## 1. Introduction

Natural products have always played a leading role, not only as a source of food and shelter for man, but also in traditional medicine to find methods to cure diseases and to extend and improve life. Considering that most of the planet surface is covered by oceans, its enormous ecosystem comprises a vast range of species, but most of them remain unidentified. To understand the magnitude of marine global species diversity, several authors reported the possible existence of 0.7 to 1.0 million marine species and about 226,000 of the described are eukaryotic [1]. Being a large ecosystem, the marine environment is considered as one of the most important sources of natural bioactive compounds with extremely rich biodiversity. Oceans cover about 71% of the earth’s surface and are mainly composed of saltwater. Sea reconnaissance as a renewable source of natural compounds has a positive impact on the development of new systems with wide biotechnical and biomedical applications [2]. When compared to bioactive compounds extracted from terrestrial life forms, marine biomaterials have shown a higher incidence of bioactivity and chemical innovation which make marine organisms an important source of structurally and biologically active secondary metabolites [3].

Over the past decades, marine-based drug discovery increased significantly, leading to at least eight related drugs approved by the United States of America Food and Drug Administration (FDA) and by the European Medicines Agency (EMA) [4]. Nowadays, there has been a growing interest in many scientific areas related to the discovery of marine compounds due to their large biodiversity and simplicity in the extraction and purification processes.

Marine biomaterials are generally biodegradable and biocompatible, and exhibit biological properties that promote the discovery of a wide range of novel bioactive compounds with specific pharmacological features of interest for the pharmaceutical industry [5,6]. Indeed, marine compounds are special because they present essential characteristics in drug discovery: good bioavailability and affinity to target [7].

Nevertheless, due to the difficulties in reproducing the marine microenvironment in the laboratory, a high percentage of marine biodiversity is still unexplored [8]. The exploitation of these compounds is still a challenge. The trend is to create new techniques for the progress of marine drug discovery and development, in particular, to improve the yield extraction, purification process, and cultivation methods [2,9,10] or implement nanotechnological approaches to overcome physicochemical issues of marine compounds. One of the most relevant characteristics of marine compounds is the fact that they are usually identified as having long aliphatic chains and several functional groups, creating a bulky hydrophobic structure, with problems of solubility [11]. This phenomenon results in low bioavailability and some new strategies need to be established to further exploit these compounds in promising applications [12].

## 2. Biomaterials as Drug Delivery Systems

A drug delivery system (DDS) can be defined as a formulation or a device that enables the introduction of a therapeutic substance in the human body [13]. Nanotechnology provided new approaches for drug delivery that improved efficacy and safety by controlling the rate, time, and place of drug release in the body. As so, the whole process involves not only the correct administration of the active substance but also its effective delivery to a specific site of action [13]. These nanometric devices act as an interface between the drug and the patient, and there is a wide spectrum of DDS to be used in nanomedicine, according to the characteristics involved in the therapy. Biomaterials are known to offer various advantages for the delivery of genetic material or therapeutic agents. Several types of DDS have been developed so far, namely, lipid-, phospholipid-, and polymer-based, which can be considered to optimize drugs’ physicochemical features as well as in vivo efficacy.

**Figure 1:**
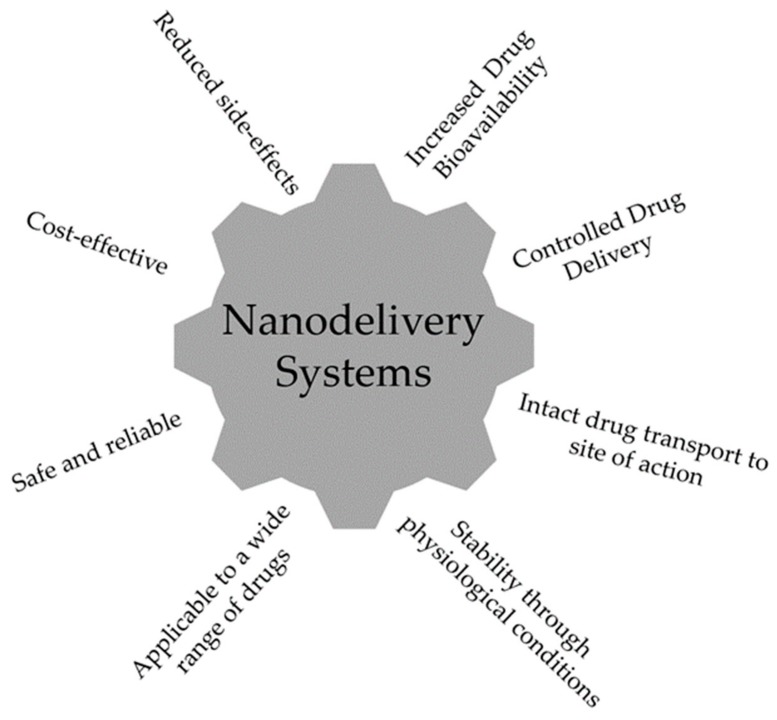
Ideal characteristics of a nanodelivery system.

The main characteristics of a nanodelivery system are illustrated in Figure 1 and these include: drug controlled release, the ability to use different routes of administration, the improved safety and efficacy of drugs, the increased solubility of low-soluble compounds, and a new market opportunity to recover drugs which failed at conventional delivery [13].

Another interesting factor is the possibility to combine diagnosis and therapy in a nanodelivery system. Indeed, nanodelivery is a very interdisciplinary area, mobilizing knowledge from subjects like chemistry, engineering, biology, and medicine [14]. Yet, nanodelivery systems are still facing some challenges mainly related to safety, efficacy, and production [13]. Studies suggest that physicochemical characteristics of a DDS determine their success in preclinical drug development, namely, the type of DDS (e.g., structure, composition, size, surface potential), the amount present in dispersion, the colloidal stability, as well as their cellular biocompatibility and interactions [15]. As a result, nanomedicine can ensure the carrying and delivery of a promising therapeutic agent to maximize its pharmacological activity and overcome possible drawbacks which may block the required effectiveness.

## 3. Marine Polysaccharides as Drug Delivery Systems

The vast marine source has proven to be valuable in different areas, such as pharmacy and cosmetics, biomedicine, and also in food science [16]. Marine biomaterials can be distributed in three main groups: polysaccharides, proteins, and lipids [9]. The focus has been given to marine polysaccharides, found in different biological sources (e.g., marine algae or animals and microorganisms), and represents a large and complex group of different macromolecules with diverse biological applications [17].

**Figure 2:**
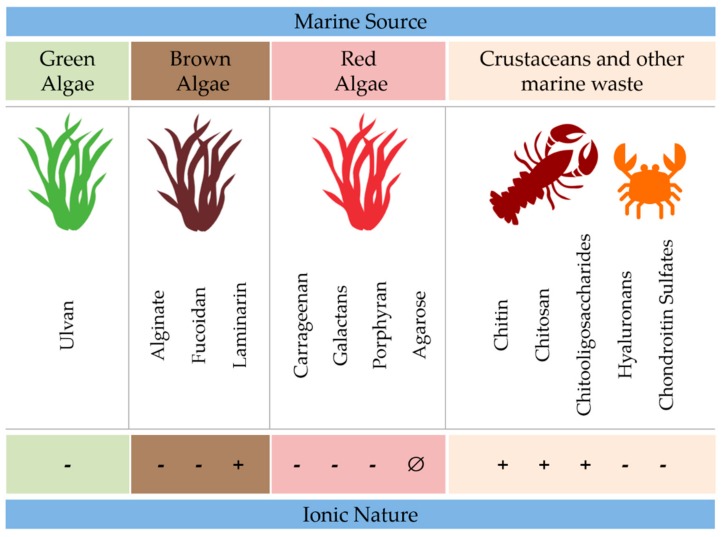
Range of marine polysaccharides available according to their source and electrostatic characteristics.

Polysaccharides are defined as polymeric carbohydrate structures formed by repeated monosaccharide units that are attached to each other by glycosidic bonds [18]. The polysaccharide classification is based on their primary or covalent structure which represents the sequence of the monomeric units along the chain. These repeated units are coupled by covalent chemical bonds which are not completely flexible, by limiting the monomers to a narrow range of orientations. Due to this characteristic, a polysaccharide chain is only able to adopt certain shapes, named “secondary structures”, depending on their primary sequence [19,20,21,22,23]. Marine polysaccharides can exhibit different chemical structures and have important biological properties such as biocompatibility, biodegradability, and anti-inflammatory activity as well as adhesive and antimicrobial activity. Properties such as shape, size, and response to stimuli are dependent on pH and temperature. Figure 2 illustrates marine polysaccharides’ allocation according to their source and electrostatic characteristics.

Moreover, marine polysaccharides have intrinsic features of great relevance in the field of drug delivery:

I Undergo chemical and enzymatic reactions to produce different materials [24];
II Are biocompatible, biodegradable, and have low immunogenic properties [22];
III Can be produced, conjugated, and complexed with proteins or other bioactive molecules [25];
IV Produce stimuli-responsive drug delivery systems [26,27];
V Can be modified as gels or give rise to interpenetrated polymeric networks [28].

### 3.1. Marine Algae

Marine algae are the major producers of all aquatic ecosystems and have served as important sources of bioactive natural substances. There are about 9000 species of macroalgae and they are classified according to their pigments: *Phaeophyta* (brown algae), *Rhodophyta* (red algae), and *Chlorophyta* (green algae). Macroalgae are a rich source of polysaccharides, minerals, vitamins, and bioactive substances used in the development of new pharmaceutical agents. One of the most studied polysaccharides from marine brown algae is fucoidan.

#### 3.1.1. Fucoidan: Sources, Chemical Structure, Biological Properties

**Figure 3:**
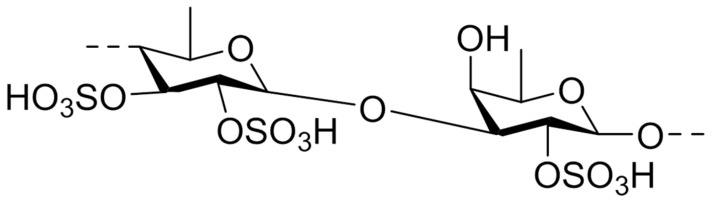
Chemical structure of fucoidan unit from *Fucus vesiculosus*.

Fucoidan was isolated for the first time in 1913 and refers to a family of sulfated polysaccharides isolated from several brown algae and marine invertebrates [29]. The structure shown in Figure 3 presents a substantial quantity of L-fucose and sulfate ester groups, but according to the source, fucoidan can exhibit different structures [30,31].

*Fucus vesiculosus* is the most common algae species used to extract the simplest polymer of the entire group having only L-fucose and sulfate units [32,33]. Structurally, fucoidan has a backbone of α-(1–3)-linked fucose units or it is composed of repeating disaccharide units of α-(1–3)- and α-(1–4)-linked fucose residues with O-2 arm. Depending on the structure of the main chain, fucoidan can be sulfonated at O-4, O-2, or at both positions of the fucose units. Beyond that, some type of fucoidan can be both sulfated and acetylated [32].

Fucoidan has been studied regarding diverse biological activities, which are related to molecular weight (M_W_), type of sugar content, sulfation degree, and molecular structure. These parameters are highly dependent on the source, harvesting, and extraction conditions. Some of the evidenced properties are antitumor, antiviral, anti-inflammatory, and a potent anticoagulant activity [16].

Despite having, as of today, a broad range of reported M_W_ (from five to several hundred kDa), Balboa et al. have suggested that low M_W_ fucoidan fractions are more biocompatible than high M_W_. The same author has identified fucoidan to exhibit some newsworthy pharmacological effects such as antithrombotic, antitumoral, antiviral, immunomodulatory, antioxidant, and anti-inflammatory activity [34]. In fact, fucoidan has a wide variety of biological activities, the anticoagulant action being the most studied. Many researchers have reported that its anticoagulant activity could be directly related to the sulfate content and position, M_W_, and sugar composition [35]. Thrombin plays an important role in thrombosis and so its inhibitor has become the main subject of studies on antithrombotic drugs. However, some researchers have reported that the anticoagulant properties of fucoidan were determined by thrombin inhibition, whose anticoagulant activity was similar to heparin [36]. In order to be able to bind the thrombin, fucoidan requires a long sugar chain and a comfortable conformation [31]. Concerning fucoidan antitumor activity and comparing with synthetic drugs, the natural products have attracted the increasing attention of patients for their biological activities and lower side effects and it has been reported that fucoidan has a cytotoxic effect via enhancing immunity on tumor cells but not on healthy cells [37].

Some authors have reported fucoidan as a pH-sensitive polymer, mainly due to the acidic functional groups in the structure and also by the total number of negatively charged groups on the chain that can urge a response to changes in external pH [38,39]. Rocha de Souza et al. have reported that fucoidan from *Fucus vesiculosus* has an inhibitory effect on the formation of hydroxyl and superoxide radicals [40]. Fucoidan from *Fucus vesiculosus* is composed of 44.1% fucose, 26.3% sulfate, and 31.1% ash [41]. This type of polymer has a relatively simple chemical composition, but most of the fucoidans have a complex composition.

**Figure 4:**
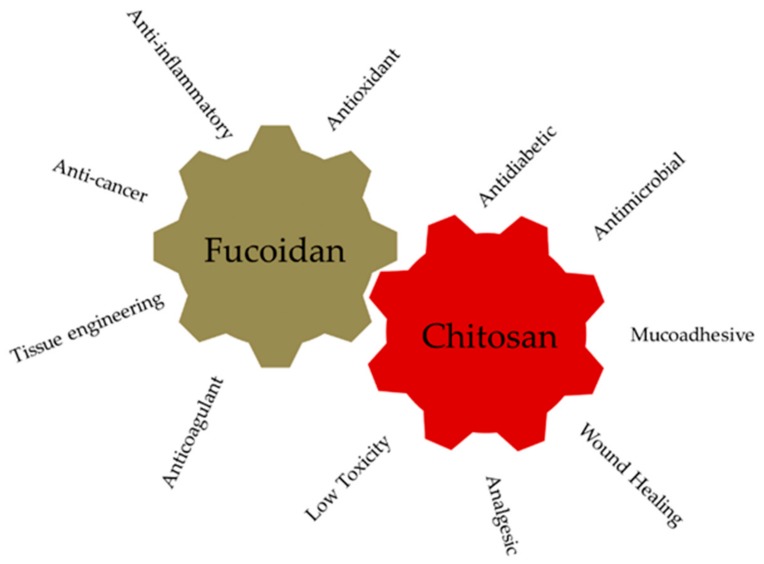
Bioactivities of fucoidan and chitosan reported in literature.

Fucoidan is an excellent drug candidate for pharmaceutical applications. Recently, fucoidan has been investigated because of its various biological properties such as anticoagulant, antiviral, antiangiogenic, antitumor, anti-inflammatory, antioxidant, antiproliferative, and immunomodulating properties (Figure 4) [31]. For example, the anticancer activity of fucoidan is mostly related to a lower M_W_. Fucoidan extracted from several species of brown algae has been characterized for its biological activities, namely, anticancer and anti-inflammatory activities (Table 1).

#### 3.1.2. Fucoidan-Based Nanoparticles and Their Applications

Fucoidan has been explored as a therapeutic compound but its unique structural features allow the design of a DDS. Chemical modifications aiming to harness specific biological activities by inducing changes in its affinity to specific drugs have increased fucoidan’s ability to encapsulate drugs increasing their release efficacy, either by chemical reactions or by interactions with other polymers [79]. Kurosaki et al. reported the first application of fucoidan as a matrix material for the synthesis of the drug delivery system regarding the delivery of protein-based drugs [80]. In this study, the positive zeta potential of the polyplexes enabled the complexation with the negatively charged fucoidan by electrostatic interactions mediated by the sulfate groups. It is possible to modulate zeta potential values in nanoparticles with sizes around 200 nm varying the used polymer ratios [81].

Fucoidan has been investigated in the biosynthesis of metal nanoparticles, such as the green synthesis of gold nanoparticles [82], and has been explored for cancer treatment. Lira and collaborators have synthesized and characterized fucoidan-coated poly (isobutyl cyanoacrylate) nanoparticles using anionic emulsion polymerization and redox radical emulsion polymerization of isobutyl cyanoacrylate. It was possible to apply fucoidan as a novel coating biomaterial, resulting in an in vitro potent cytotoxic activity against macrophage and fibroblast cell lines [83]. Research groups reported the biosynthesis of silver nanoparticles using carboxymethylated curdlan or fucoidan as reducing and stabilizing agents, showing potent in vitro anticancer activity against osteosarcoma [83].

### 3.2. Marine Crustaceans

Crustaceans, including crayfish, crab, shrimp, and lobster, are a class of species that is widely explored in the seafood industry. Seafood processing involves the extraction of heads, bones, exoskeleton, and carapace of the described animals, which produces a large amount of waste. This bioprocess industry represents a good opportunity to convert waste into the extraction of compounds of interest for possible biomedical applications, and at the same time solve the pollution problem related to seafood processing. One of the most explored polysaccharides from marine crustaceans is chitosan.

#### 3.2.1. Chitosan: Sources, Chemical Structure, Biological Properties

**Figure 5:**
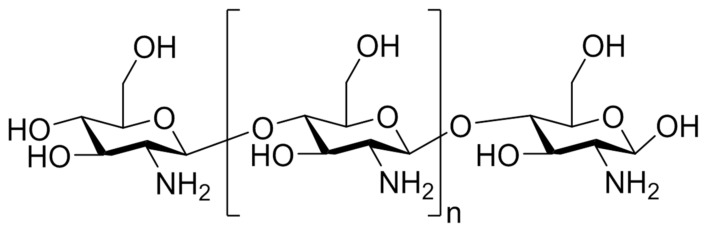
Chemical structure of chitosan.

Chitosan was first reported in 1884 and it is a linear polysaccharide which is obtained by the deacetylation of chitin, found mainly in the exoskeleton of arthropods and crustaceans (Figure 5) [84].

The acetylation degree reflects the balance between the two types of residues and differentiates chitin from chitosan. When, after the deacetylation process, the outcome has a molar percentage lower than 50% mol, the product is named chitosan and becomes soluble in acidic aqueous solutions [85]. Chitosan physical properties depend on the degree of acetylation and on the acetyl group distribution along the chains. The presence of amine groups makes it positively charged in acidic environments and neutral in alkaline pH values due to a pKa value close to 6 [86]. The interesting characteristics of chitosan rely on its cationic nature and high charge density in solution, which confers pH responsiveness and mucoadhesive properties [87]. This marine polysaccharide is one of the most abundant and it is widely used and studied for biomedical applications such as antimicrobial activity in wound infection, and antitumor and anti-inflammatory activities (Figure 4) [88].

#### 3.2.2. Chitosan-Based Nanoparticles and Their Applications

Considering its properties, chitosan is an excellent material to use in biological environments and to design drug delivery systems. Chitosan can be chemically modified to alter its functionality. As a biomaterial, it is able to perform controlled drug release of cationic drugs; has mucoadhesive properties due to the presence of cationic primary amino groups; allows the preparation of hydrogels; forms stable complexes with large polyanionic molecules such as DNA-based drugs; and allows permeation enhancement because it interacts with cell membrane, promoting a reorganization of tight junction-associated properties [89]. Chitosan exhibits many beneficial properties such as biocompatibility, biodegradability, safety, and relevant biological activities which lead to intense research for biomedical applications [5].

Regarding oral delivery, the high solubility of chitosan at acidic pH values is a major drawback. Concerning its cationic nature and high charge density in a solution, chitosan forms stable ionic complexes with multivalent water-soluble polyanions under mild physiological conditions [90,91]. Mucoadhesive polymers have been usually applied to the fabrication of nanoparticles (NPs) for oral administration [92,93]. In particular, chitosan enhances paracellular drug transport via a transient opening of the tight junction between epithelial cells [94,95,96]. The mucous membrane or mucous layer consists of mucous-secreting epithelial cells and is present on the inner side of many organs of the human body, such as in the gastrointestinal tract (GIT) and upper respiratory tract. Water makes up more than 95% of the total mucous weight, making it very hydrated, and its thickness varies with the region. Most of the polymers that interact with mucins in the gut are hydrophilic and positively charged in the gut environment. Chitosan has hydroxyl and amine groups that can give rise to hydrogen bonding-mediated interactions with the components of the mucus and has been studied for mucosal drug delivery, namely, oral [96], buccal [97], and nasal [98,99]. The positive charges on the chain can interact with sialic and sulfonic acids of the mucus layer by strong electrostatic interactions, and therefore exhibit favorable cell adhesion due to the attraction to the negative charge of the cell membrane [99]. Considering all the advantages of using biodegradable and biocompatible mucoadhesive polymers, the ones that stand out exhibit increased residence time in the intestine and thus prolonged contact with the intestinal surface membranes, which leads to an improvement in the absorption [100].

In addition to its mucoadhesive properties, chitosan has attracted much interest also for its ability to act as permeation enhancers and as efflux transporter, P-glycoprotein inhibitor, already demonstrated in vitro and in vivo [101]. Chitosan polymer also exhibits good antimicrobial and antioxidant properties, a broad spectrum of activity, and lower toxicity toward several cell lines [102]. In particular, chitosan-based nanoparticles, especially the low-M_W_ ones, can penetrate the bacteria cell wall, combine with DNA, and inhibit mRNA synthesis and DNA transcription [103,104]. On the other hand, high-M_W_ chitosan-based nanoparticles can interact with the cell surface and consequently alter cell permeability [105]. Similarly, for chitosan antioxidant activity, researchers have described an antioxidant action by scavenging oxygen radicals such as hydroxyl as well as highly stable 2,2-diphenyl-1-(2,4,6-trinitrophenol)hydrazyl (DPPH) radicals. Park et al. have also demonstrated that low-M_W_ chitosan is more active than high-M_W_ [106].

As a DDS, chitosan can be used as a stable solid dosage form in oral delivery, as hydrogels, nanoparticles, and colloidal systems to apply by ophthalmic route; to give mucoadhesive properties in nasal route spray formulations and buccal drug delivery; to confer polymer robustness to vaginal tablets; if it is purified, to enable use by parenteral route because it is nontoxic; and also to combine antigens to produce vaccines [89].

Chitosan nanoparticles can be produced by different methods (ionotropic gelation, microemulsion method, emulsification solvent diffusion method, polyelectrolyte complexes, and reverse micellar method [107]) and are used in a wide array of applications, relying on their excellent physicochemical properties and bioactivity without harming the human organism. These nanoparticles are used in the field of tissue engineering (e.g., design of implants), cancer therapy (e.g., in theranostic applications and drug delivery), enzyme immobilization support, encapsulation of biologically active compounds, and as an antimicrobial agent [107]. Given chitosan’s anti-inflammatory activity, chitosan biomedical applications include wound healing and antibacterial and hypocholesterolemic effects. Chitosan may induce analgesia in wound management, giving a cool and soothing effect, and this also happens when applied as a topical treatment of burns, skin abrasions, skin ulcers, and skin grafted areas [108].

## 4. Fucoidan–Chitosan Nanoparticles

Given the abundant presence of fucoidan and chitosan, as well as their biodegradability, biocompatibility, and bioactivity, application in drug delivery systems leads to the production of nanoparticles as well as other delivery platforms, namely, hydrogels and scaffolds. The electrostatic interactions between positively charged chitosan and negatively charged fucoidan can be explored to produce different types of nanoparticles using mainly the polyelectrolyte self-assembly and the ionotropic crosslinking methodologies. In the following sections, the production methods and the developed fucoidan–chitosan nanoparticles, as well as other types of delivery systems found in the literature, will be reviewed.

### 4.1. Preparation Methods of Fucoidan–Chitosan Nanoparticles

**Figure 6:**
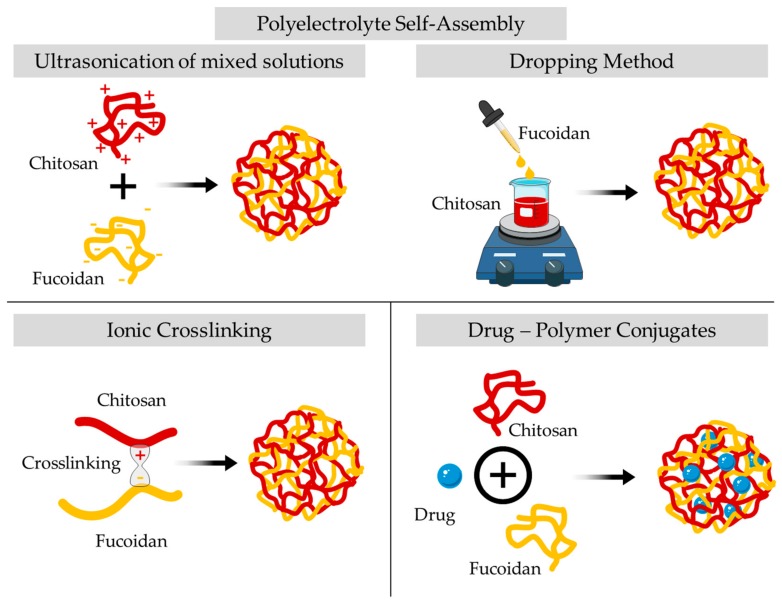
Schematic representation of the diverse methods described for the preparation of fucoidan–chitosan nanoparticles.

Fucoidan–chitosan nanoparticles can be obtained by complexation or conjugation methods (Figure 6).

The most commonly used preparation strategy is complexation by employing a simple polyelectrolyte self-assemble method initially described by Lee et al. [109]. In this procedure, it is possible to combine different weight ratios by mixing solutions of chitosan to solutions of fucoidan, and then the mixture is sonicated at room temperature [110,111,112,113,114]. According to the literature, with this method, it is possible to obtain nanoparticles of 100–300 nm with approximately 90% encapsulation efficiency. In the optimization of fucoidan–chitosan nanoparticles production, the effect of the pH level of chitosan solution and the fucoidan–chitosan mass ratio was investigated using the dropping method [115]. The nanoparticles tended to grow in size as the pH of chitosan was increased up to 3.69, after which they became smaller. This pattern of growth is prominent as the mass ratio of fucoidan and chitosan increases. The authors suggested a pH 5 and a mass ratio of 1:1 to obtain a high yield in nanoparticles of small size and with good suspension stability. Fucoidan–chitosan nanoparticles can also be complexed by the dropping method. This method involves the dropwise addition of different fucoidan solutions into the chitosan solution under continuous stirring over 30 min, to achieve the nanoparticles [116,117,118,119]. Usually, this protocol leads to nanoparticles with a size range from 356 to 900 nm.

The preparation of fucoidan–chitosan nanoparticles can be achieved by the ionic crosslinking method due to ionic bonds linking one polymer chain to another. In this procedure, solutions of fucoidan are added to chitosan solutions by flush mixing using a pipette tip with ultrasonic vibration in an ice bath [120,121,122,123]. Through this technique, it is also possible to achieve entrapment efficiencies of 90%, with sizes up to 500 nm due to a wider entangling of the molecular chains. Liu and coworkers studied the influence of chitosan M_W_ on the fucoidan nanocarriers obtained by ionic crosslinking [110]. When the M_W_ increased from 50 to 150 kDa, the particle size decreased while the uniformity improved. The ionic crosslinking between fucoidan and chitosan is governed by the increase in chitosan’s M_W_ since it increases the binding sites and leads to a more compact structure and subsequent decrease of the particle size. A qualitative approach was designed based on a relative charge density model of the fucoidan–chitosan nanoparticles to establish the extent of the ionic interactions in terms of polyelectrolyte complexes [116]. Likewise, a quantitative approach was set up based on a relative charge density model from the stoichiometric distributions of both positive amino groups and negative sulphate ions loaded in fucoidan–chitosan nanoparticles to predict their preparation conditions [117].

Fucoidan–chitosan nanoparticles for drug delivery can also be attained with drug–polymer conjugates. In this case, the polymers that constitute the nanoparticle are conjugated with the drug by direct covalent linkage or noncovalent interactions, and generally, this conjugation occurs by a stable or cleavable linker, especially if the main goal of the drug is to reach the intracellular target of the conjugate [118].

Of notice is that the production of nanoparticles composed of the same polysaccharides with different synthesis methods results in different physicochemical properties, giving these nanosystems a broad range of applications in pharmaceutics but also in cosmetics and food products.

### 4.2. Nanomedicine Applications of Fucoidan–Chitosan Nanoparticles

Interactions between the amino group of chitosan and the sulfate group of fucoidan, ionized under acidic conditions, allow the formation of nanoparticles and limit drug release [110]. Given the deprotonation of the chitosan’s amino groups at pH 7.4, electrostatic interactions weaken and the nanoparticles swell and disintegrate to release the loaded drug, which may hamper fucoidan–chitosan nanoparticles’ therapeutic applications [110,111]. However, the pH-responsive profile of fucoidan–chitosan nanoparticles prevents degradation under acidic conditions of the gastrointestinal tract and allows drug absorption in the intestine. Hence, fucoidan–chitosan nanoparticles have been widely explored for oral delivery of active pharmaceutical ingredients [110,111,113,118,123,124,125,126,127,128,129], also taking advantage of chitosan’s property of increased contact with the mucus layer and consequently longer resistance times at the absorption site [129]. The biological properties of fucoidan described in Section 3.1.1 were explored by some authors to obtain synergic effects with a drug, for example, ciprofloxacin [117] or gemcitabine [126].

Huang and collaborators have designed and evaluated fucoidan–chitosan nanoparticles using the polyelectrolyte self-assembly method combined with ultrasonication at room temperature as an oral delivery system [111]. The nanoparticles obtained with fucoidan from *Fucus vesiculosus* were stable at pH 2.5 and decomposed at pH 7.4. In vitro data from an intestinal model revealed the ability of the fucoidan–chitosan nanoparticles to effectively enhance the opening of the cell tight junction, demonstrating their potential application as carriers for oral delivery.

#### 4.2.1. Polyphenolic Compounds Delivery

Curcumin, a polyphenolic compound from the herb *Curcuma longa*, has a variety of biological activities described including its potential for antitumor activity [130]. Yet, the poor solubility of curcumin in aqueous solution and its rapid degradation by enzymes in the intestinal tract hampers its bioavailability and clinical efficacy. Huang et al. have used a simple polyelectrolyte self-assembly method under ultrasonication at room temperature to incorporate curcumin in the fucoidan–chitosan nanoparticles. The structures with a size range between 200 and 300 nm at pH 1.2 became larger and unstable as the pH increased. By adjusting the weight ratio of chitosan to fucoidan, nanoparticles can be made pH-sensitive and suitable for use as oral delivery carriers. In vitro drug release data indicated that the curcumin encapsulated in nanoparticles could be prevented from deterioration when passing through the stomach, and then effectively released in the intestine, increasing the absorption and bioavailability of curcumin. Yet, the amount of curcumin released at pH 2.5, the fasting pH value in the stomach, was higher than at pH 1.2, indicating that the fucoidan–chitosan nanoparticles were not ideal for fasting in the oral system [110]. To overcome this issue, chitosan solubility was increased by introducing a carboxymethyl group and then the O-carboxymethyl chitosan was applied in the design of an oral delivery system for curcumin, upon complexation with fucoidan in the presence of calcium ions. O-carboxymethyl chitosan–fucoidan nanoparticles were obtained and the encapsulated curcumin was stable in a simulated pH environment (pH 2.5). The encapsulated drug was less cytotoxic toward fibroblasts than the free form and was internalized by intestinal Caco-2 cells through energy-dependent endocytic pathways, suggesting fucoidan–chitosan nanoparticles’ promising application as carriers in oral delivery systems [118]. Huang and coworkers have follow-up research with a similar carrier like the ones described above, designed for oral delivery of soluble eggshell membrane proteins toward the therapy of defective intestinal epithelial cells. In this work, the research group demonstrated targeted delivery, mucoadhesiveness, and controlled release to intestinal epithelial cells [112].

Another approach for oral delivery of hydrophobic and hydrophilic drugs was designed with arginine-modified chitosan and thiolated fucoidan to enhance the transport of dextran and curcumin across the intestinal barrier [124]. The nanoparticles exhibited a pH-sensitive assembly–disassembly and drug release property. However, the cationic amino acid, arginine, on the carrier led to disruption of intestinal epithelial tight junctions and, consequently, the permeation studies revealed that the nanoparticles enhanced the paracellular permeation of macromolecular dextran through the intestinal monolayer. The aqueous solubility of curcumin was improved with the encapsulation in the thiolated-fucoidan/arginine–chitosan nanoparticles, thus enhancing its intestinal permeability.

Moreover, the benefits of fucoidan–chitosan nanoparticles in polyphenols delivery by improving aqueous solubility and, consequently, oral bioavailability, led also to the development of nanostructures for the delivery of berberine, red ginseng, and quercetin [113,127,131]. Generally, these nanoparticles demonstrated their ability to control polyphenols release, retain biological activity, and prevent degradation. Structural changes were made to fucoidan by conjugation with taurine, that had a sulfonate group of taurine and thus increased the negative-charge density on fucoidan. This approach allowed the production of a fucoidan–taurine-shelled nanoparticle containing berberine and the amelioration of defective intestinal epithelial tight junction barrier caused by bacterial endotoxin. The nanoparticles were effective in local berberine delivery with controlled release able to restore barrier function in inflammatory and injured intestinal epithelial [113]. Kim et al. explored the bioactivity of fucoidan for improving the antithrombotic activity of red ginseng and demonstrated significant effects in vivo of the anticoagulation activity of fucoidan and significantly lower thrombus formation than free red ginseng [131]. A rationale based on different weight ratios of fucoidan and chitosan was applied to obtain pH-responsive nanoparticles to improve the oral bioavailability of quercetin [127]. Fucoidan–chitosan nanoparticles with higher amounts (3/1 and 5/1) of fucoidan from *Fucus vesiculosus* (M_W_ 50–190 kDa, pKa 1.0–2.5) were resistant to the gastrointestinal pH environments and retained quercetin antioxidant activity.

#### 4.2.2. Antibacterial Agents Delivery

In an attempt to combine antimicrobial and antioxidant functions into a drug delivery system to treat pneumonia infection, Huang and Li have developed fucoidan–chitosan nanoparticles for pulmonary delivery of gentamicin [112]. At first, chitosan was treated with H_2_O_2_ to produce low-M_W_ chitosan (38 kDa). The simple polyelectrolyte self-assembly method was used to produce the nanoparticles with various chitosan-to-fucoidan weight ratios, using low-M_W_ chitosan (38 kDa, upon treatment with H_2_O_2_). The fucoidan–chitosan nanoparticles at 5/1 ratio remained stable for 25 days in phosphate-buffered saline (pH 6.0–7.4) and exhibited a controlled release of gentamicin for up to 72 h. The developed nanoparticles exerted no cytotoxic effects on lung cells and did not induce unwanted inflammatory reactions while exhibiting scavenging effects and antioxidant activities. As a follow-up, the same research group described a biphasic release of gentamicin from fucoidan–chitosan nanoparticles produced by ionotropic crosslinking [120]. Here, the nanoparticles exhibited a zero-order release of gentamicin for the first 10 h, followed by a sustained release of up to 72 h. Pharmacokinetics data indicated that encapsulated gentamicin can effectively improve antimicrobial activity and reduce systemic toxicity. Although the preparation of fucoidan–chitosan nanoparticles was similar in the previous study (i.e., ionotropic crosslinking), a different group of nanoparticles was developed to deliver ciprofloxacin toward intracellular *Salmonella* bacteria, responsible for food-born infections and commonly associated with biofilms infections [117]. Initially, ciprofloxacin-loaded chitosan nanoparticles were obtained by the ionic crosslinking method using tripolyphosphate as a crosslinker, and after sonication, the mixture was stirred with an aqueous fucoidan solution. Encapsulated ciprofloxacin has better antimicrobial activities than the free drug, and can enhance biofilm eradication efficiency higher than the bare drug. These outcomes of the developed delivery systems can be related to (i) fucoidan’s ability to interact with the scavenger receptor and thus enhance intracellular delivery of the nanoparticles to macrophages; and (ii) fucoidan and chitosan antibacterial activities which act in synergy with ciprofloxacin.

Of notice is the importance of M_W_ in the properties of polymers; for example, chitosan with lower M_W_ (ca. 5 kDa) can pass through the microbial membrane because of its small size and solubility in water [132,133] while low-M_W_ fucoidan shows higher anticoagulation activity and higher antioxidative activity via scavenging superoxide/hydroxyl radicals and inhibiting low-density lipoprotein oxidation than high-M_W_ fucoidan [17,134]. Tsai and collaborators have exploited the antibacterial and antioxidant activities of low-M_W_ fucoidan and chitosan upon the formation of a covalently linked conjugate [135]. The polymer conjugates synthesized by amidation form colloidal nanoparticles and exhibit higher DPPH and superoxide anion radical scavenging activities than low-M_W_ chitosan alone. Also, antibacterial activity toward *Escherichia coli* and *Staphylococcus aureus* indicated that the conjugation of both low-M_W_ polymers improved the bacterial inhibition of fucoidan alone.

#### 4.2.3. Tissue Engineering Applications

Angiogenesis involves the formation of new blood capillaries and is required for physiological processes, including embryonic development, tissue regeneration, and wound repair, and is also required for invasive tumor growth and metastasis. Angiogenesis inhibitors target the tumor microenvironment through inhibiting the growth of the neovasculature. As previously listed, among several biological activities, fucoidan has the ability to inhibit angiogenesis. Given the fact that sulfate content and M_W_ influence the anticoagulant and angiogenic activities of fucoidan, oversulfated fucoidan was exploited in inhibiting the basic fibroblast growth factor (bFGF) or vascular endothelial growth factor (VEGF)-induced vascular tube formation [121,123]. Oversulfated fucoidan–chitosan nanoparticles were prepared by a polycation/polyanion self-assembly method and were able to improve intestinal paracellular transport, probably related to a transient opening of the tight junctions of intestinal cells [123]. Moreover, the pH-sensitive characteristics of the designed nanoparticles could be applied as a pH-switched nanocarrier for oral delivery of the antiangiogenic macromolecule in response to simulated gastrointestinal tract media. Yu and coworkers demonstrated that the transported oversulfated fucoidan significantly inhibited the tube formation of endothelial cells. In a different approach toward angiogenesis control, bFGF was loaded in fucoidan–chitosan nanoparticles by ionic crosslinking for nerve tissue engineering [123]. The research group demonstrated the importance of the weight ratio of fucoidan:chitosan in the nanoparticles’ release profile and protective effect of bFGF. Also, since the release from the nanoparticles was slow, the carriers significantly decreased the amount of bFGF needed for neurite extension.

Huang and Liu have also investigated the applicability of fucoidan–chitosan nanoparticles in the tissue engineering field [119]. Stromal cell-derived factor 1 (SDF-1) was incorporated in fucoidan–chitosan nanoparticles by ionotropic crosslinking with tripolyphosphate, which was able to protect SDF-1 against proteolysis, pH variation, and heat. A controlled release of the chemokine was observed for up to seven days and in vitro data on mesenchymal stem cells confirmed SDF-1 mitogenic activity and the ability to promote migration and to regulate signaling pathways. Although no further studies were found in the literature, the marine-origin delivery systems demonstrated promising results in delivering a chemokine for tissue regeneration.

Considering fucoidan anticoagulant activity, Silva and collaborators have explored a fucoidan-based nanosystem for oral administration with an antithrombotic effect [125]. To confer higher gastric pH resistance to nanoparticles, the strategy employed glutaraldehyde crosslinks between chitosan chains. Fucoidan release assay showed that at a sustained release at pH 7.4 over time, the fucoidan–chitosan nanoparticles significantly reduced thrombus formation in the deep vein thrombosis model and showed better behavior than fucoidan.

#### 4.2.4. Anticancer Compounds Delivery

In an effort to treat cancer, a marine-origin drug delivery system based on fucoidan and chitosan has been developed aiming more effective and tolerated therapies toward breast [126] and prostate [136] cancer. Oliveira and coworkers have optimized the production of fucoidan–chitosan nanoparticles by polyelectrolyte complexation, using fucoidan from *Fucus vesiculosus* (M_W_ 45–75 kDa) and chitosan (M_W_ 40–150 kDa) [126]. An increase in the stability of the nanoparticles was evident upon crosslinking with 1-[3-(dimethylamino)-propyl]-3-ethylcarbodiimide hydrochloride (EDC) and N-hydroxysuccinimide (NHS). Encapsulated gemcitabine was released from the marine-origin polymeric nanoparticles at 37 °C in a sustained profile for up to 4 h, then maintained a steady-state profile for up to 72 h. This nanocarrier increased toxicity toward human breast cancer cells when compared with free gemcitabine, without increasing their toxic effect on endothelial cells. Another approach, involving the ionic gelation method, led to the incorporation of piperlongumine within fucoidan–chitosan nanoparticles [136]. Encapsulation of this pro-oxidant drug efficiently killed prostate cancer cells mediated by intracellular reactive oxygen species generation and did not demonstrate cytotoxicity toward human fibroblasts. This cancer-specific anticancer effect will be further exploited using chemical modifications in the marine-origin complex to avoid the nonspecific bindings of plasma proteins, which is essential to enhance their in vivo pharmacokinetic properties.

#### 4.2.5. Coating of Metallic Nanoparticles

In nanomedicine, increasing interest has been observed in the application of metallic nanoparticles (e.g., gold, silver, or iron oxide) for imaging diagnosis and even therapy, as it allows external control of drug delivery. To ensure metallic nanoparticles’ stability in the biological environment, the coating is essential. This process can be performed during or after the synthesis of the metallic nanoparticles, or these carriers can be encapsulated inside a nanostructure material (e.g., a liposome or micelle).

Venkatesan and coworkers applied fucoidan in the green synthesis of silver nanoparticles (AgNPs), and further coated them with chitosan to form an electrolyte complex on the surface [137]. Fourier transform infrared spectroscopy data suggested strong polyelectrolyte complexation between fucoidan and chitosan. The developed chitosan–fucoidan complex-coated AgNPs significantly inhibited microbial growth and exhibited efficient anticancer activity in human cervical cancer cells (HeLa).

As an alternative to conventional cancer therapy, photothermal therapy using gold nanoparticles has been explored. In this approach, chitosan/fucoidan multilayer coatings of gold nanorods were synthesized and evaluated owing to their good biocompatibility, photostability, and strong absorption in the near-infrared (NIR) region. The in vitro photothermal efficiency of the multifunctional carriers using laser irradiation at 1.0 W/cm^2^ for 5 min was promising and further assessed in vivo. The tumor in a xenograft model after treatment with the multifunctional carriers and laser irradiation had almost completely disappeared [138].

#### 4.2.6. Other Applications

An attempt to develop fucoidan–chitosan nanoparticles for topical delivery of an anti-inflammatory agent took advantage of the effect of polymer weight ratio [139]. Methotrexate was loaded within the marine-origin nanoparticles following a polyelectrolyte self-assembly method using ultrasonication at room temperature. Encapsulated methotrexate in 3/1 and 5/1 ratio nanoparticles did not affect fibroblasts and keratinocytes viability and presented lower cytotoxicity than free methotrexate. The fucoidan–chitosan nanoparticles retained the anti-inflammatory activity of methotrexate and improved skin permeation in relation to the free drug.

Given the significant mucoadhesive and absorption-enhancing properties of trimethyl chitosan and the hypoglycemic effects of fucoidan, Tsai and coworkers have developed a multifunctional nanoparticle to deliver insulin by oral administration [140]. The developed nanoparticles protected insulin against degradation by releasing insulin in a pH-dependent manner in the gastrointestinal tract fluids and were able to modulate the barrier function of the Caco-2 intestinal epithelial cell monolayer, with an enhancement in the paracellular transport of insulin across the intestinal barrier. The authors expect that the absorption enhancement observed, combined with the biological effect of fucoidan, could advance the development of multifunctional therapeutic platforms for diabetes.

### 4.3. Biomedical Applications of Fucoidan and Chitosan

In tissue engineering, biomaterials play a crucial role in providing a template and extracellular environment to support regenerative cells and promote tissue regeneration. Fucoidan has important properties for artificial bone development, namely, inducing osteogenic genes and enhancing febrile collagen matrix formation, while chitosan can be used to modify cell adhesion, proliferation, and differentiation. Scaffolds composed of fucoidan and chitosan have been established as biomaterials for bone regeneration [141,142,143,144,145,146]. Likewise, several examples of hydrogels based on fucoidan and chitosan can be found in the literature developed mainly as a solution for wound healing [144,147,148,149,150]. This research interest is closely related to the known biological activities of the marine-based polymers, in particular, chitosan, which can be applied as dressing for proliferation and activation of inflammatory cells [151], and fucoidan, which exhibits significant gel contraction-promoting, integrin expression-enhancing, and heparin activities [152], revealing that both are good players to design wound-healing solutions.

## 5. Conclusions

Marine organisms represent an important source of polysaccharides suitable for biomedical applications. Natural biomaterials like fucoidan and chitosan exhibit important physicochemical and biological properties with recognized therapeutic effects. Yet, certain limitations such as the purification and the hydrophilic nature still require improvements. Diverse studies have been made to employ fucoidan and chitosan as nanoparticles for pharmaceutical applications, namely, for exploiting many biological drugs that have poor aqueous solubility and permeability, and less bioavailability. Chemical modifications can be performed to improve bioavailability or bioactivity of these marine-origin polysaccharides per se. Although these polysaccharides present specific individual characteristics, their combination has been exploited in order to improve their biomedical applications. These specific fucoidan–chitosan nanostructures have already proven to be helpful in drug delivery, but also in the biocompatibility enhancement of other designed systems, such as nanoparticles, hydrogels, or other designed scaffolds. The application of fucoidan and chitosan together in nanostructures appears to be a promising approach for future developments in the biomedical field.

## Figures and Tables

**Table 1:** Sources of fucoidan and their described biological activities.

Fucoidan Source	Biological Activity	
	Anticancer	Anti-Inflammatory
*Cladosiphon okamuranus*	[41,42]	[43]
*Cladosiphon okamuranus Tokida*	[44]	[45]
*Costaria costata*	[46]	[47]
*Ecklonia cava*	[46]	[48]
*Eisenia bicyclis*	[49]	[50]
*Fucus evanescens*	[51,52,53]	[54]
*Fucus vesiculosus*	[41,55,56,57,58,59,60,61,62,63]	[64,65,66,67]
*Fucus sp.*	[68]	[69]
*Laminaria japonica*	[70]	[66,67]
*Macrocystis pyrifera*	[71]	[66,67]
*Sargassum sp.*	[68]	[72]
*Saccharina japonica*	[73]	[74]
*Turbinaria conoides*	[75]	[76]
*Undaria pinnatifida*	[51,73,77]	[78]
